# Therapeutic preference for Alzheimer’s disease treatments: a discrete choice experiment with caregivers and neurologists

**DOI:** 10.1186/s13195-023-01207-8

**Published:** 2023-03-24

**Authors:** George Dranitsaris, Quanwu Zhang, Lin Mu, Christopher Weyrer, Erik Drysdale, Peter Neumann, Alireza Atri, Amir Abbas Tahami Monfared

**Affiliations:** 1 Department of Public Health, Falk College, Syracuse University, 150 Crouse Dr., Syracuse, NY 13244 USA; 2 Eisai, Inc., Nutley, NJ USA; 3 Boston Consulting Group, Boston, MA USA; 4 Tufts Medical Center, Boston, MA USA; 5 Banner Sun Health Research Institute, Sun City, AZ USA; 6 Brigham and Women’s Hospital and Harvard Medical School, Boston, MA USA; 7 McGill University, Montreal, QC Canada

**Keywords:** Alzheimer’s disease, Caregiver preference, Physician preference, Discrete choice experiment

## Abstract

**Background:**

Alzheimer’s disease (AD) is a major global health crisis in need of more effective therapies. However, difficult choices to optimize value-based care will need to be made. While identifying preferred therapeutic attributes of new AD therapies is necessary, few studies have explored how preferences may vary between the stakeholders. In this study, the trade-offs among key attributes of amyloid plaque-lowering therapies for AD were assessed using a discrete choice experiment (DCE) and compared between caregivers and neurologists.

**Methods:**

An initial pilot study was conducted to identify the potentially relevant features of a new therapy. The DCE evaluated seven drug attributes: clinical effects in terms of delay in AD progression over the standard of care (SOC), variation in clinical effects, biomarker response (achieving amyloid plaque clearance on PET scan), amyloid-related imaging abnormalities-edema (ARIA-E), duration of therapy, need for treatment titration as well as route, and frequency of drug administration. Respondents were then randomly presented with 12 choice sets of treatment options and asked to select their preferred option in each choice set. Hierarchical Bayesian regression modeling was used to estimate weighted preference attributes, which were presented as mean partial utility scores (pUS), with higher scores suggesting an increased preference.

**Results:**

Both caregivers (*n* = 137) and neurologists (*n* = 161) considered clinical effects (mean pUS = 0.47 and 0.82) and a 5% incremental in ARIA-E (mean pUS =  − 0.26 and − 0.52) to be highly impactful determinants of therapeutic choice. In contrast, variation in clinical effects (mean pUS = 0.12 and 0.14) and treatment duration (mean pUS =  − 0.02 and − 0.13) were the least important characteristics of any new treatment. Neurologists’ also indicated that subcutaneous drug delivery (mean pUS = 0.42 vs. 0.07) and administration every 4 weeks (mean pUS = 1.0 vs. 0.20) are highly desirable therapeutic features. Respondents were willing to accept up to a 9% increment in ARIA-E for one additional year of delayed progression.

**Conclusions:**

Caregivers and neurologists considered incremental clinical benefit over SOC and safety to be highly desirable qualities for a new drug that could clear amyloid plaques and delay clinical progression and indicated a willingness to accept incremental ARIA-E to achieve additional clinical benefits.

**Supplementary Information:**

The online version contains supplementary material available at 10.1186/s13195-023-01207-8.

## Introduction

Dementia is a clinical condition characterized by the progressive deterioration of at least two cognitive domains such as memory, language, personality, behavior, or visuospatial and executive function [1]. Alzheimer’s disease (AD) is the most common type of dementia, accounting for approximately 80% of all new diagnoses [2]. AD is a disease primarily diagnosed in the elderly, and the prevalence is expected to increase with the aging global population [2]. Indeed, the incidence of AD is expected to rise twofold every 5 years in people 65 years and older [3]. In the United States of America (USA) alone, approximately 5.8 million Americans above 65 years of age have AD [4]. Furthermore, the number of cases in the USA is expected to rise to 13 million by 2050 as the elderly population increases to 88 million [4]. This represents a major concern for health policy decision-makers and for the allocation of societal resources. In the USA, the direct costs of AD medical management and the indirect costs of lost productivity are estimated to be $305 billion annually [5]. Similar prevalence and cost estimates have also been reported for Europe [6].

The natural history of clinical progression in AD is characterized by a gradual decline, within and across multiple domains, as the disease advances through mild, moderate, and severe stages [1]. Impairment is higher with moderate to severe disease with patients demonstrating a decline in physical functioning (e.g., apraxia; impaired body coordination with frequent falls and uncontrolled sphincters) and in cognitive functioning (e.g., memory loss, speech difficulties, and disorientation in space and time) [1, 7]. Such declines severely impact patient quality of life (QOL) and well-being [8]. There is also a tremendous burden on the caregiver [7]. One study from Portugal evaluated depression, anxiety, and stress in 102 caregivers of AD patients using validated instruments [9]. The investigators determined that number of days of care and duration of daily care for AD patients severely impacted caregivers’ QOL. Furthermore, caregivers supporting a patient with more memory and behavioral problems reported greater stress, depression, and anxiety compared to those who were supporting patients with less advanced disease [9].

To mitigate clinical decline and support QOL, the current standard of care in AD is to augment behavioral, environmental, and lifestyle management strategies (a.k.a. non-pharmacological management) and management of co-morbidities (e.g., hypertension, sleep apnea), with stage-dependent symptomatic pharmacotherapy using FDA-approved anti-AD medications. The two main classes of drugs first used to manage AD were the cholinesterase inhibitors (i.e., donepezil, rivastigmine, and galantamine) and the *N*-Methyl-d-aspartate receptor antagonists (e.g., memantine) [1, 10]. Despite the availability of these drugs, the clinical benefit derived by patients is limited. To provide more effective agents, there are several targets in the disease pathophysiology such as the brain-derived neurotrophic factor, nerve growth factor, and gene therapies that are attractive in terms of drug development [10]. Indeed, the current AD drug development pipeline is rich with over 140 different agents that focus on an array of complementary mechanisms and targets. Of these developmental agents, over 80% are considered disease-modifying treatments (DMTs), and the most studied DMTs have targeted reduction in toxic amyloid beta (AB) species via various mechanisms including inhibition of enzymes that facilitate the formation of toxic amyloid species, vaccination, and immunotherapy using monoclonal antibodies (mAbs) that target various amyloid species [10–12]. Unfortunately, many of the initial mAb trials did not meet their primary endpoints for multiple reasons such as inappropriate study designs, inadequate study models, and poor patient selection [10, 13]. However, the insights gained from these failed trials are being used to better guide drug development. Indeed, newer agents such as aducanumab, donanemab, lecanemab, gantenerumab, and solanezumab hold considerably more promise, and clinical trial results in multiple AD populations are pending for several of these agents [14].

Given the number of promising agents currently in phase 2 and 3 development, including several amyloid plaque-lowering mAb trials nearing readout in late 2022 and early 2023, there is a rational basis for hope that, in the coming years, targeted agents may be available for the treatment of AD. Such a scenario would necessitate therapeutic choices by key stakeholders such as caregivers, patients, neurologists, and other treating clinicians, as well as drug formulary committees. One approach to measure individual preferences and the relative value of multiple attributes in the utility of a drug product or health service is through a discrete choice experiment (DCE) [15, 16]. More commonly used in the transport and environmental economics literature, DCE is a stated preference technique designed to identify and measure the relative importance of each product attribute and the strength of influence of each level within that attribute [17]. Through the application of DCE and hierarchical Bayesian analyses, the aim of the current study was to assess caregiver and neurologist treatment preferences and trade-offs among seven key therapeutic attributes of AD amyloid plaque-lowering therapies.

## Methods

### Designing a discrete choice experiment

A DCE is a quantitative method used to elicit preferences from respondents who are directly interested in the drug or healthcare service under investigation [15]. A DCE typically begins with an initial pilot study in a small sample of respondents to help identify the attributes that may be most relevant [16, 17]. The results from the pilot study are then used to develop the DCE survey instrument, which can have multiple levels within each attribute. The final sample of participants is then presented with a series of alternative hypothetical choice set scenarios containing some or all the pilot-tested attributes, where each may have several levels. Respondents are then asked to indicate their preferred choice between two or more alternative options, each presented at one of its levels [16, 17]. In most DCE surveys, respondents are typically presented with no more than 12 choice experiments to avoid cognitive overload. However, a recent survey of DCE studies revealed that approximately 29% of respondents answered 8 or fewer choice sets and about 15% answered more than 16 choice sets [18].

Through this approach of obtaining the favored choices, preferences are determined without explicitly asking respondents to state their preferred level for each individual attribute under investigation. To obtain the final preference estimates for each attribute (known as partial utilities or partworth), multivariable multinomial regression modeling is typically used.

In this DCE, we created a full-profile, twelve-choice set, two-alternative, seven-attribute, forced-choice, main effect, fractional factorial design, using D(B)-optimal design for the multinomial logit model with the idefix package in R [19, 20]. A total of 12 unique choice sets were generated and presented to each respondent in a random order. The seven attributes consisted of average clinical effects (i.e., average years of delay in progression to moderate stage of AD), variation in clinical effects (i.e., distribution in the magnitude of clinical effects achieved among patients), biomarker response (proportion of patients achieving amyloid clearance on PET scan at 1 year), symptomatic amyloid-related imaging abnormalities-edema (ARIA-E) as a known side effect of amyloid-targeting therapies, duration of therapy, need for titration at treatment initiation, and treatment administration characterized by route and frequency (Table 1). For each of these seven attributes, the D(B)-optimal design specified its direction of utility and type of coding: positive priors for average clinical effects and biomarker response, negative priors for symptomatic ARIA-E, and non-informative priors for the remaining attributes; continuous coding for numerical attributes and dummy coding for categorical attributes. Of note, our choice for continuous (vs. dummy) coding for numerical attributes reflected a preference to prioritize design efficiency across all attributes over the ability to test non-linear utilities of numerical attributes; we deprioritized non-linearity tests, because we did not find evidence of a material non-linear utility function within the attribute levels that were chosen for this DCE, that were informed by late (vs. early) stage clinical trial data.

**Table 1:** Attributes and attribute levels presented for hypothetical treatments choice set scenarios

Therapeutic attributes	Levels
**Clinical effects (average):** average years of delay in progression to moderate stage of Alzheimer’s disease, compared to standard of care	• 1.5 years • 2.5 years • 3.5 years
**Clinical effects (variation):** distribution in the magnitude of clinical effects achieved among patients	• Narrow • Wide
**Biomarker response (amyloid clearance):** proportion of patients achieving amyloid negativity on PET scan at 1 year	• 3 in 10 patients (30%) • 7 in 10 patients (70%)
**Adverse events (symptomatic ARIA-E):** proportion of patients developing symptomatic ARIA-E during treatment	• 1 in 100 patients (1%) • 1 in 20 patients (5%) • 1 in 4 patients (25%)
**Treatment duration:** total length of entire treatment course	• 1 year • 3 years • 5 years
**Treatment titration (at initiation):** requirement to adjust dosage of medication when first starting treatment	• Does not require dosage titration • Requires dosage titration (typically for 3–8 months)
**Treatment administration:** route and frequency of medication administration	• Intravenous every 2 weeks • Intravenous every 4 weeks • Subcutaneous every 2 weeks

*ARIA-E* Amyloid-related imaging abnormalities-edema

### Target population

The primary data for the DCE were obtained via an online survey. The survey population consisted of two samples from the USA, caregivers who care for family members or friends with clinically diagnosed AD and neurologists who actively treat patients with AD. Access to these individuals was obtained from a database of neurologists and AD caregivers managed by an external vendor. Invitations were sent to neurologists and caregivers to participate in the survey. Respondents were provided background information on the intent of the study, and all provided written informed consent to participate. Respondents were also informed that complete confidentially will be maintained and their personal information would not appear in any study report or publication. The study protocol received ethical approval from Pearl IRB (Indianapolis, IN; IRB protocol # 22-BCGR-101). All subjects provided informed consent to participate in the survey. The study was conducted in accordance with the Declaration of Helsinki.

### Questionnaire development and administration

Questionnaires were developed to initially capture respondent demographic data. Information on caregivers consisted of sex, age group, race, ethnicity, education, annual household income, and caregiving experience (e.g., AD stage of patients under care, type of care provided, duration and frequency of caregiving, cohabitation with patients). Demographic data on the neurologists’ sample consisted of practice setting, years of practice, AD patient volume, and prior use of aducanumab.

**Fig. 1:**
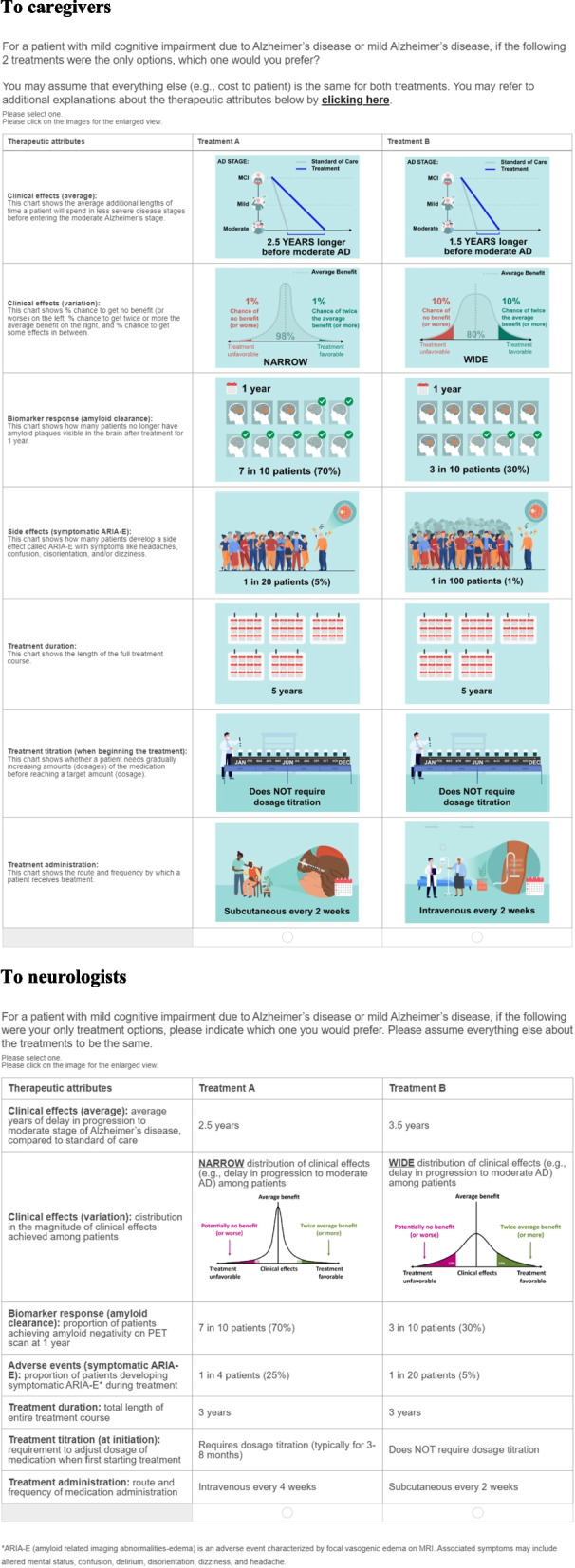
Example of a discrete choice a question presented to each respondent cohort

The second part of the questionnaires consisted of a choice between two hypothetical treatment choice set scenarios where respondents were asked to indicate their therapeutic preference for patients with early AD, which was based on the most relevant attributes identified after the series of pilot interviews. The relevant attributes were selected through qualitative interviews of a pilot sample of caregivers and neurologists using a series of open-ended questions to identify those most relevant in guiding treatment choice. Respondents were told to assume that other decision factors (e.g., out-of-pocket costs to patients, QOL gains) were equal between the choices presented. Each participant was presented with 12 randomized choice sets, plus two internal validity test sets, one repeating a previous set to assess the stability of the respondents’ preference and the other that was a dominated pair to assess the attention or comprehension of each respondent (Fig. 1). A total of seven therapeutic attributes composed of multiple levels were evaluated (Table 1). A utility is a generic preference-based score, with higher scores indicating greater preferences for that attribute. Each attribute evaluated in a DCE is presented as a partial utility. An overall utility score for a given therapeutic choice in a DCE consists of the sum of all product attributes. For each of the therapeutic attributes in the current study, the outcomes were presented as a mean partial utility where a larger value suggested a greater average preference for the cohort under investigation.

### Statistical analysis

Respondent characteristics were summarized descriptively as means, medians, or proportions, with appropriate levels of variance (i.e., standard error (SE), standard deviation (SD), and 95%CI). Partial utilities of the various therapeutic properties of competing alternatives were generated using hierarchical Bayesian (HB) models, with random effects coefficients. The HB model attributes’ priors and coding are consistent with their D(B)-optimal design and unstructured covariance which makes no explicit assumption on the covariance between attributes. Markov chain Monte Carlo (MCMC) simulations were used with 3 chains, a MCMC sample size of 25,000, a burn-in period of 200,000, and a thinning interval of 2. Of note, a large burn-in was used to enable model convergence. The HB models were conducted through the bayesmh command in STATA, version 16.0 [21].

In the primary analyses, the mean (SE) and SD (SE) for the partial utilities were estimated for each attribute at the population level. The means were generated using the average of respondent-level partial utilities, weighted by the inverse posterior variance to account for observed heteroskedasticity; such a weighting reduced the potential bias due to larger uncertainty associated with larger respondent-level partial utility estimates. The SDs were estimated using the average of SDs associated with the respondent-level partial utilities. The SEs were estimated using 500 bootstraps of respondent-level partial utility means or SDs. Preference heterogeneity for each therapeutic attribute was assessed using the coefficient of variation (CV) for the partial utility at the population level. A sensitivity analysis was conducted to assess the partial utility estimates only among respondents who (1) demonstrated stable preferences (i.e., consistently chose the same option in the same repeated choice set) and (2) did not demonstrate attribute dominance in choosing alternatives (i.e., always chose alternatives based only on average clinical effects).

The means (SEs) for the relative importance of each attribute at the population level were also estimated using 500 bootstraps of population-level partial utilities, that were then used to calculate the ratios between the partial-utility range of each attribute and the sum of the partial-utility ranges of all attributes at prespecified levels. Of note, the prespecified levels were not defined simply as the highest and lowest levels of each attribute (e.g., for average clinical effects, a 1-year increment was chosen over a 2-year increment); their selection considered profiles of the late-stage and post-approval amyloid-targeting agents that caregivers and neurologists may encounter in the clinical practice.

In economics, the marginal rate of substitution (MRS) is the rate at which a unit of an item can be replaced by another while providing the same level of satisfaction to the consumer [17]. For example, a respondent may be willing to accept additional ARIA-E events in exchange for an additional year of delayed AD progression. The MRS is calculated by dividing the HB model coefficients of two competing numerical attributes of interest. The medians with 95%CI for the MRS of the numeric attributes (ARIA-E events, biomarker response, and treatment duration) in exchange for a 1-year increment of clinical effects at the population level were estimated from 500 bootstraps of the respondent-level partial utility ratios between clinical effects and the numeric attribute of interest, weighted by the average inverse posterior variance of the two attributes. All the statistical analyses were performed using STATA, version 16.0 [21].

## Results

A sample of 291 caregivers and over 200 neurologists were approached, and 180 and 179 provided consent, respectively, to participate in the study. Of the 180 caregivers and 179 neurologists who completed the online survey, 43 and 18 were excluded, respectively, from the analyses due to inadequate attention or based on failing an a priori set of comprehension-based criteria (e.g., failure to choose the clearly superior treatment alternative in a test choice set). No difference in respondent characteristics was observed between the initial sample and retained sample of respondents (*p* > 0.35 for all chi-square tests by respondent characteristics, data not shown).

Respondent characteristics for caregivers and neurologists are presented in Tables 2 and 3, respectively. In the caregiver sample, approximately 64.9% of the sample was female and over 50% was older than 55 years of age (Table 2). Most of the sample was white (86.1%), and 35% have an annual household income of $100,000 or more. Furthermore, over 50% of caregiver respondents had at least a bachelor’s degree, and 33 of 137 (24%) had experience as a healthcare professional. Most of the caregiver cohort (97.8%) was a family member of an AD patient(s), and 122 of 137 respondents (89.1%) indicated that at least one of the patients in their care had moderate or severe disease (Table 2). The type of care provided was broad, from self-care activities to the management of behavioral symptoms. Overall, 46% of caregivers provided AD patient support six or more times per week, and 64.9% of the sample had been doing this for at least 3 years (Table 2). Among neurologist respondents, 37.3% practiced in an academic center, 23.0% in a rural setting, 42.2% had over 20 years of practice experience, 46.6% had treated AD patients within the last 12 months, and nearly one-third had experience using aducanumab (Table 3).

**Table 2:** Characteristics of AD caregivers in primary analyses

Caregiver characteristic	*N* = 137 (%)
Female gender	89 (64.9)
Age	
18 to 34 years old	20 (14.6%)
35 to 44 years old	26 (18.9%)
45 to 54 years old	20 (14.6%)
55 to 64 years old	43 (31.4%)
65 years or older	28 (20.4%)
Race^a^	
White	118 (86.1%)
Black or African American	7 (5.1%)
Asian	5 (3.7%)
Others	7 (5.1%)
Annual household income	
$34,999 or lower	24 (17.5%)
$35,000 to $49,999	12 (8.8%)
$50,000 to $74,999	27 (19.7%)
$75,000 to $99,999	22 (16.1%)
$100,000 or higher	48 (35.0%)
Decline to answer	4 (2.9%)
Educational attainment	
Less than high school	4 (2.9%)
High school diploma	13 (9.5%)
Some college or associate degree	39 (28.5%)
Bachelor’s degree	43 (31.4%)
Some graduate school or graduate degree	38 (27.7%)
Experience as a healthcare professional	33 (24.0%)
Relationship of patient(s) to caregiver participants	
Family member	134 (97.8%)
Friend	3 (2.2%)
Alzheimer’s disease stage of patient(s) under care^a^	
Mild cognitive impairment	104 (75.9%)
Mild stage	103 (75.2%)
Moderate or severe stage	122 (89.1%)
Type of care provided^a^	
Support basic self-care activities: e.g., bathing, feeding	77 (56.2%)
Support key day-to-day activities: e.g., shopping, housekeeping	122 (89.1%)
Manage behavioral symptoms: e.g., wandering, agitation, anxiety	82 (59.9%)
Find and use support services: e.g., paid in-home aides	71 (51.8%)
Duration of caregiving experience	
Less than 1 year	12 (8.8%)
1–2 years	36 (26.3%)
3–4 years	49 (35.7%)
5–6 years	17 (12.4%)
More than 6 years	23 (16.8%)
Frequency of care provided	
Less than 1 time a week	7 (5.1%)
1–2 times a week	24 (17.5%)
3–5 times a week	43 (31.4%)
6 or more times a week	63 (46.0%)
Live together with patient(s) under care	
Yes	87 (63.5%)

^a^*N* (%) of subgroups by characteristic do not add up to 137 (100%) because respondents were allowed to make multiple selections in the survey

**Table 3:** Characteristics of neurologists in primary analyses

Neurologist characteristic	*N* = 161 (%)
Setting of clinical practice(s)^a^	
Solo practice	21 (13.0%)
Neurology group practice	64 (39.8%)
Multispecialty group practice	26 (16.2%)
Academic medical center	60 (37.3%)
Urbanity of clinical practice(s)^a^	
Rural (nonmetro)	37 (23.0%)
Urban (metro)	126 (78.3%)
Geographic region of clinical practice(s)	
Northeast	50 (31.1%)
Midwest	23 (14.3%)
South	53 (32.9%)
West	35 (21.7%)
Years of clinical practice in neurology	
0–4 years	10 (6.2%)
5–9 years	20 (12.4%)
10–14 years	26 (16.2%)
15–19 years	37 (23.0%)
20 + years	68 (42.2%)
Number of Alzheimer’s disease patients treated last year	
0–24 patients	12 (7.5%)
25–49 patients	17 (10.6%)
50–74 patients	31 (19.3%)
75–99 patients	26 (16.2%)
100 + patients	75 (46.6%)
Experience with aducanumab	47 (29.2%)

^a^*N* (%) of subgroups by characteristic do not add up to 161 (100%) because respondents were allowed to make multiple selections in the survey

**Fig. 2:**
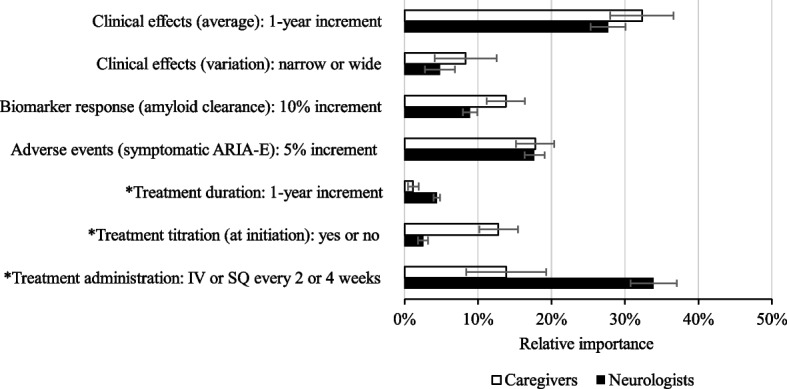
Relative importance of therapeutic attributes to caregivers and neurologists. *Abbreviations*: ARIA-E, amyloid-related imaging abnormalities-edema; IV, intravenous; SC, subcutaneous. The data are presented as means with standard errors (SE)

The first series of outcomes evaluated from the two respondent groups was the relative importance of the various therapeutic attributes described in Table 1. As illustrated in Fig. 2, both caregivers and neurologists considered ARIA-E events (presented in 5% increments) and average clinical effects (presented as 1-year increments for a delay in the progression of AD to a moderate stage) to be very important therapeutic attributes (combined relative importance: 58% for caregivers, 50% for neurologists). In contrast, variation in clinical effects (i.e., a wide vs. narrow distribution in the magnitude of clinical effects achieved among patients) and treatment duration were the least important therapeutic attributes. It was also interesting to note that between the groups, caregivers valued the need for treatment titration significantly more than neurologists (13% vs. 3%, *p* < 0.05). In contrast, neurologists valued treatment administration (every 4 vs. every 2 weeks) significantly more than caregivers (34% vs. 14%, *p* < 0.05).

**Fig. 3:**
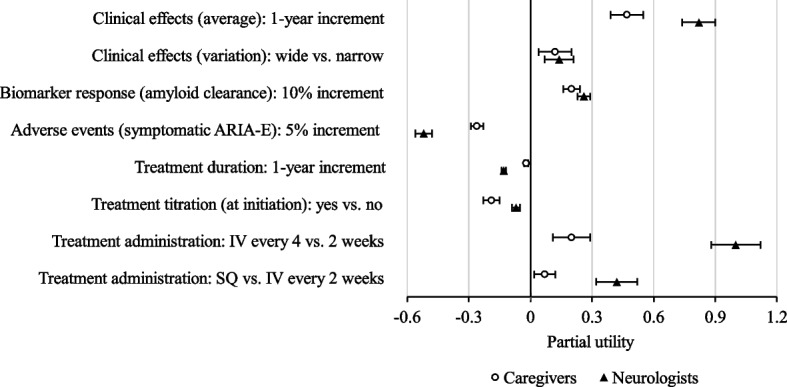
Partial utilities of therapeutic attributes among caregivers and Neurologists^1^. *Abbreviations*: ARIA-E, amyloid-related imaging abnormalities-edema; IV, intravenous; SC, subcutaneous. ^1^A larger partial utility indicates a greater impact on preferences: a positive partial utility indicates a more preferred/favorable attribute, while a negative partial utility indicates a less preferred/favorable attribute. Partial utilities should not be interpreted by themselves but compared between attributes within a respondent group. Error bars represent the standard errors of the mean partial utilities. The data are presented as means with standard errors

The analysis was continued with an evaluation of the partial utilities generated from the statistical analysis. The means and SDs for the partial utility of each attribute among caregivers and neurologists are presented in Table 4 and Fig. 3. Consistent with the findings from the preference assessment, the average clinical effects, presented as 1-year increments for a delay in the progression of AD compared to the current standard of care (SOC), had a high mean partial utility for both caregivers and neurologists (Fig. 3). Similarly, increments in ARIA-E events relative to the SOC were associated with the highest partial disutility score (or a negative partial utility score) for both caregivers and neurologists (Table 4). However, with both of these attributes, the neurologist scores were approximately twofold higher than those from caregivers (Fig. 3). Furthermore, neurologists had significantly higher partial utilities for a treatment administered subcutaneously (SC) or intravenous (IV) every 4 weeks. However, partial utilities were comparable between the respondent groups with respect to variation in treatment effects (wide vs. narrow) and biomarker response (i.e., the proportion of patients achieving amyloid clearance on PET scan at 1 year). The sensitivity analysis conducted among respondents who demonstrated stable preferences and did not rely on a dominant strategy in choosing alternatives (*N* = 100 for caregivers, *N* = 126 for neurologists) revealed stability in the primary findings (see table supplement). Of note, a comparable pattern and magnitude in the SD and CVs were seen between the main and sensitivity analyses, suggesting preference heterogeneity caused by true diversity in respondent preferences. In summary, on average, caregivers and neurologists preferred longer-lasting clinical effects, fewer ARIA-E events, stronger biomarker response, and less frequent intravenous treatment.

**Table 4:** Partial utilities of therapeutic attributes among caregivers and neurologists

Therapeutic attribute	Caregivers (*N* = 137)		Neurologists (*N* = 161)	
	^a^**Mean (SE)**	**SD (SE)**	^**a**^**Mean (SE)**	**SD (SE)**
Clinical effects (average): 1-year increment	0.47 (0.08)	0.65 (0.01)	0.82 (0.08)	0.70 (0.01)
Clinical effects (variation): wide vs. narrow	0.12 (0.08)	0.70 (0.01)	0.14 (0.07)	0.75 (0.01)
Biomarker response (amyloid clearance): 10% increment	0.20 (0.04)	0.28 (< 0.01)	0.26 (0.03)	0.24 (< 0.01)
Adverse events (symptomatic ARIA-E): 5% increment	− 0.26 (0.03)	0.25 (< 0.01)	− 0.52 (0.04)	0.41 (0.01)
Treatment duration: 1-year increment	− 0.02 (0.01)	0.13 (< 0.01)	− 0.13 (0.01)	0.15 (< 0.01)
Treatment titration (at initiation): yes vs. no	− 0.19 (0.04)	0.57 (< 0.01)	− 0.07 (0.02)	0.35 (< 0.01)
Treatment administration: IV every 4 vs. 2 weeks	0.20 (0.09)	0.83 (0.01)	1.00 (0.12)	1.16 (0.02)
Treatment administration: SC vs. IV every 2 weeks	0.07 (0.05)	0.72 (0.01)	0.42 (0.10)	1.10 (0.01)

*ARIA-E* Amyloid-related imaging abnormalities-edema, *IV* Intravenous, *SD* Standard deviation, *SE* Standard error, *SC* Subcutaneous

^a^A utility is a preference-based score, with higher scores indicating higher preferences for that attribute. For each attribute, a larger mean partial utility indicates a greater average preference in the sample, and a larger SD partial utility indicates greater heterogeneity in preference within the sample

The final evaluation was the estimation of the MRS to determine what respondents were willing to trade for one additional year of delayed AD progression. Table 5 presents the MRS for these clinical attributes. In exchange for 1 additional year of delayed progression, neurologists were willing to trade a larger reduction in biomarker response than caregivers (a 38% vs. 20% reduction in amyloid negativity at 1 year). However, both neurologists and caregivers were willing to accept an increment in symptomatic ARIA-E events, 7 and 9% respectively, for an additional year of clinical benefit. Similarly, both groups were willing to accept a longer treatment duration (approximately 6 years) for that additional year of delayed AD progression from a mild to a moderate stage of disease (Table 5).

**Table 5:** Marginal rates of substitution for clinical effects

In exchange for 1 additional year of delayed progression, respondents are willing to trade	MRS for caregivers, median (95%CI)	MRS for neurologists, median (95%CI)
Biomarker response (amyloid clearance)	20% (16–27%)	38% (27–45%)
Adverse events (symptomatic ARIA-E)^a^	9% (8–11%)	7% (5–11%)
Treatment duration	6.3 (4.6–8.6) years	5.6 (4.8–7.2) years

*ARIA-E* Amyloid-related imaging abnormalities-edema, *MRS* Marginal rate of substitution, reported as median (95% confidence interval)

^a^In the case of tradeoffs for adverse events, this is also known as the maximum acceptable risk (MAR), which indicates a patients’ willingness to trade off harmful and beneficial outcomes from a given treatment

## Discussion

This current study used a DCE to evaluate the relative importance of seven primary attributes in the provision of new amyloid plaque-lowering therapies for AD and to estimate what a subset of key stakeholders, caregivers to individuals with AD, and treating neurologists would be willing to trade off as measured by their marginal rate of substitution. Not surprisingly, the findings suggest that both caregivers and neurologists were most predisposed to a new AD amyloid plaque-lowering drug that offered robust clinical benefit in terms of delaying clinical progression, with a low frequency of ARIA-E and a convenient administration schedule. Therapeutic characteristics deemed to be of lesser importance by both respondent groups consisted of variation in clinical effects (i.e., the distribution in the magnitude of clinical effects achieved among patients), biomarker response, duration of therapy, and the need for treatment titration. This was illustrated, for example, by the observations that respondents were willing to trade 20 to 38% in biomarker response of amyloid PET negativity, to accept a duration of therapy of 6 years, in order to gain an additional year of delayed clinical progression.

The therapeutic attributes in which divergence was seen between caregivers and neurologists consisted of frequency and method of drug delivery, where neurologists had stronger preferences for SC or IV administration every 4 weeks as opposed to IV administration every 2 weeks. Indeed, neurologists considered IV administration every 4 weeks of greater value than an additional year of progression-free benefit. Caregivers and neurologists also considered one additional year of delayed progression sufficient to accept a 7 to 9% increase in symptomatic ARIA-E.

The variation noted in the clinical effect outcomes may be due to different interpretations by the key stakeholder groups of the efficacy data reported in clinical trials. It is tempting to speculate that such preference heterogeneity could suggest that some caregivers may favor, or weight more heavily, the value of hope in their therapeutic decision-making, while some neurologists, such as those with prior experience of aducanumab use, may potentially prioritize clinical effects generated by well-designed randomized trials, or potentially reflect their negative sentiments with respect to the inconsistent phase 3 trial results seen with aducanumab [22, 23].

A review of the AD literature revealed a paucity of utility data from the various stakeholders’ groups on the different aspects of AD pharmacotherapy. As several large registration trials evaluating amyloid plaque-lowering mAb (e.g., lecanemab, gantenerumab, donanemab) approach completion in 2022 and 2023, preference information from the key stakeholders can help inform availability, coverage, accessibility, and health policy decision making. To our knowledge, this is the first study to identify and quantify, via a systematic process and comparative analyses, the various attributes of amyloid plaque-lowering therapies for AD. The results of this investigation merit further study, replication, and assessment in a broader population of important stakeholders, especially individuals with early-stage AD. Nonetheless, these results can begin to fill the knowledge gap in the literature and to help inform clinical and health policy decision-making when assessing the value of emerging amyloid plaque-lowering therapeutics for AD. The study revealed, and quantified, that amyloid plaque-lowering AD drug delivery attributes such as the frequency and route of administration are also highly valued by AD caregivers and treating neurologists in addition to trade-offs regarding health outcomes and symptomatic ARIA-E side effects.

The strengths of this study are the inclusion of two important groups involved in AD patient care that were selected using a systematic approach. Still, there are several limitations that need to be acknowledged. First and foremost, patients with early-stage AD, who would be potential candidates for these emerging amyloid plaque-lowering therapies, were not included in the study. The sample size of both respondent groups was small and was limited to the USA and to neurologists—and not other clinicians who treat AD including primary care providers, geriatricians, and psychiatrists. This limits the generalizability of the findings to other AD-treating clinicians and to similar stakeholders in other countries, particularly in those with socialized healthcare systems and a formal health technology assessment process for new drug reimbursement submissions. Another limitation that restricts external validity and warrants further study in a more diverse and representative US patient population because 86% of our sample of AD caregiver respondents were white and 51% had attained a bachelor’s or advanced degree. The DCE methodology measures intent based on hypothetical choice set scenarios and may not necessarily reflect the true preferences of caregivers and neurologists when faced with an actual situation when such a decision needs to be made. Respondents were told to assume that other decision factors (e.g., out-of-pocket costs to patients, QOL gains) were equal between the choices presented. This is a simplified assumption and may not be realistic across the different healthcare jurisdictions and drugs currently under development. Given the risk of cognitive overload, the DCE methodology is limited in the number of attributes that can be investigated; therefore, some important attributes such as patient QOL improvements and the avoidance of patient injuries secondary to a response to treatment were not included. Finally, the survey was administered using an online format, which can also produce a selection bias towards younger respondents or those with easier access or a greater affinity to the Internet.

## Conclusions

This paper described the application of a DCE technique to measure caregivers’ and neurologists’ preferences for the various characteristics of new amyloid plaque-lowering therapeutics for early AD. For patients with early-stage AD, caregivers and neurologists from the USA had a similar relative preference for amyloid plaque-lowering therapeutics in terms of clinical effectiveness at an acceptable level of ARIA-E events as well as a more convenient every 4-week administration frequency. Both respondent groups indicated that the duration of therapy, amyloid negatively on PET biomarker after 1 year of treatment, and the need for treatment titration to be of lower priority. These results require further study, replication, and generalization in key stakeholders, most notably patients with early-stage AD, but may preliminarily assist clinicians, health policy, and drug formulary committees regarding relative value-weighting of additional attributes of emerging amyloid plaque-lowering AD therapies beyond clinical effectiveness and safety considerations.

## Supplementary Information

**Additional file 1: Table S1.** Sensitivity Analyses on Partial Utilities of Therapeutic Attributes Among Caregivers and Neurologists. Abbreviations: ARIA-E, amyloid-related imaging abnormalities-edema; IV, intravenous; SD, standard deviation; SE, standard error; SQ, subcutaneous.

## Data Availability

All data and study materials are available upon request to the corresponding author.

## Abbreviations

AB: Amyloid beta
AD: Alzheimer’s disease
ARIA-E: Amyloid-related imaging abnormalities-edema
CV: Coefficient of variation
DCE: Discrete choice experiment
HB: Hierarchical Bayesian
IV: Intravenous
MRS: Marginal rate of substitution
mAbs: Monoclonal antibodies
QOL: Quality of life
SOC: Standard of care
SE: Standard error
SD: Standard deviation
SC: Subcutaneously
