# Supplementary material for: Therapeutic preference for Alzheimer’s disease treatments: a discrete choice experiment with caregivers and neurologists
Source: Alzheimers Res Ther. 2023 Mar 24;15:60. doi: 10.1186/s13195-023-01207-8 (PMC10037811; doi:10.1186/s13195-023-01207-8)
Supplement: Supplementary file 1 — Additional file 1: Table S1. Sensitivity Analyses on Partial Utilities of Therapeutic Attributes Among Caregivers and Neurologists. Abbreviations: ARIA-E, amyloid-related imaging abnormalities-edema; IV, intravenous; SD, standard deviation; SE, standard error; SQ, subcutaneous. [file 13195_2023_1207_MOESM1_ESM.docx]

**Supplemental Table. Sensitivity Analyses on Partial Utilities of Therapeutic Attributes Among Caregivers and Neurologists**

| Therapeutic attribute | Caregivers (N=100) | | Neurologists (N=126) | |
| --- | --- | --- | --- | --- |
|  | Mean (SE) | SD (SE) | Mean (SE) | SD (SE) |
| Clinical effects (average): 1-year increment | 0.46 (0.08) | 0.65 (0.01) | 0.78 (0.08) | 0.70 (0.01) |
| Clinical effects (variation): wide vs. narrow | 0.17 (0.09) | 0.71 (0.01) | 0.24 (0.08) | 0.76 (0.01) |
| Biomarker response (amyloid clearance): 10% increment | 0.27 (0.05) | 0.29 (0.01) | 0.30 (0.03) | 0.24 (<0.01) |
| Adverse events (symptomatic ARIA-E): 5% increment | -0.32 (0.04) | 0.26 (<0.01) | -0.60 (0.05) | 0.43 (0.02) |
| Treatment duration: 1-year increment | -0.04 (0.01) | 0.13 (<0.01) | -0.15 (0.01) | 0.16 (<0.01) |
| Treatment titration (at initiation): yes vs. no | -0.22 (0.04) | 0.57 (<0.01) | -0.06 (0.02) | 0.35 (<0.01) |
| Treatment administration: IV every 4 vs. 2 weeks | 0.27 (0.10) | 0.84 (0.01) | 1.24 (0.13) | 1.18 (0.02) |
| Treatment administration: SQ vs. IV every 2 weeks | 0.001 (0.06) | 0.73 (0.01) | 0.49 (0.11) | 1.11 (0.01) |

Abbreviations: ARIA-E, amyloid-related imaging abnormalities-edema; IV, intravenous; SD, standard deviation; SE, standard error; SQ, subcutaneous
